# Wild edible plants collected and consumed by the locals in Daqinggou, Inner Mongolia, China

**DOI:** 10.1186/s13002-020-00411-2

**Published:** 2020-10-09

**Authors:** Sachula, Geilebagan, Yan-ying Zhang, Hui Zhao, Khasbagan

**Affiliations:** 1 College of Life Science and Technology, Inner Mongolia Normal University, Hohhot, 010022 People’s Republic of China; 2 Institute for the History of Science and Technology, Inner Mongolia Normal University, Hohhot, 010022 People’s Republic of China

**Keywords:** Wild edible plants, Mongolian people, Han Chinese, Daqinggou, CFSI, Ethnobotany

## Abstract

**Background:**

Knowledge of wild edible plants is an important part of traditional knowledge. It is closely related to traditional human agriculture, as well as biodiversity. This study aimed to conduct a detailed investigation and evaluation of wild edible plants that are collected and consumed by the Mongolian and Han locals in Daqinggou and to provide valuable data for the development and utilization of plant resources.

**Methods:**

In the 9 site visits to the area of Daqinggou during the period of 2017–2019, the authors used key informant interviews, semistructured interviews, and questionnaires to collect utilization information regarding precollected species of local wild edible plants. By combining the data obtained from 101 key informants, the authors used the Cultural Food Significance Index (CFSI), a quantitative index to evaluate the relative importance of the wild edible plants that were discussed in the aforementioned interviews.

**Results:**

The investigation results show that the Mongolian people provided 67 folk names, corresponding to 57 wild plants, and the Han Chinese provided 58 folk names, corresponding to 49 wild plants. A total of 61 edible wild plant species belonging to 29 families and 52 genera were recorded as edible resources for the locals in Daqinggou. The uses include grains, oil and fat resources, vegetables, fruits, beverages, condiments, and snacks. The most commonly reported purpose of wild edible plants is using them as vegetables, followed by using them as beverages and fruits. The most widely used edible parts are fruits, leaves, and other aerial parts. Eating raw and cooked plants are the usual methods of consuming wild edible plants according to the locals. In addition, the CFSI of 61 wild edible plant species shows that 27 species have characteristics of medical food.

**Conclusions:**

The knowledge and experience of naming and consuming wild plants by the Mongolian people and Han Chinese in Daqinggou are an important manifestation of the direct interaction between locals and plants. The CSFI evaluation of the wild edible plants consumed by the locals in Daqinggou establishes the utilization of some wild plants as part of the traditional knowledge of medical food.

## Background

Since 1992, as party to the Convention on Biological Diversity (CBD), China has made positive efforts in the protection and inheritance of traditional knowledge related to biological diversity and biological resources. The diversity of traditional edible plant resources and related knowledge are important aspects of traditional knowledge and biodiversity. They have a significant impact on the conservation and sustainable development of regional biodiversity [1–6]. As most kinds of edible plants are wild plants, research on wild edible plants is also an important subject of ethnobotanical study [7].

Wild edible plant species are uncultivated and undomesticated. They can be food sources, however, through collection and consumption from the natural environment. Many linguistic groups refer to numerous edible plants as “famine relief food,” which plays an important role in the survival of individuals or the whole community during a period of food shortage [8]. In recent years, wild edible plants have become popular for their nutritional value and health care functions [9]. Therefore, the utilization of wild edible plants is continuously increasing. The ethnobotany research in China concerning wild edible plants has so far been concentrated mainly in South China. For example, the Naxi people, Hani people, and Tibetan people in Yunnan Province have the habit of gathering and eating wild plants [10–12]. The Shui people and Dong people of Guizhou Province have recorded the use of wild plants as starters for preparing fermented beverages [13, 14]. Although some papers on the use of wild food plants have been published from the area of northern China (e.g., Inner Mongolia [15, 16], Tibet [17], Shaanxi [18, 19], and Gansu [20, 21]), this area is not widely studied.

The Inner Mongolia Autonomous Region is multiethnic, with Mongolian culture as the main body and Han Chinese as the majority. Since the 1990s, ethnobotanists have carried out a series of studies on regional ethnobotany in several regions of Inner Mongolia and have cataloged the wild edible plants of the local Mongolian people [15, 16, 22–24]. In the past, ethnobotanical studies in Inner Mongolia have not been performed with comparative studies between Mongolian people and Han Chinese. This study investigates the Mongolian people and Han Chinese in Daqinggou and records the wild edible plants and related traditional knowledge that locals have previously used and are currently using.

**Fig. 1:**
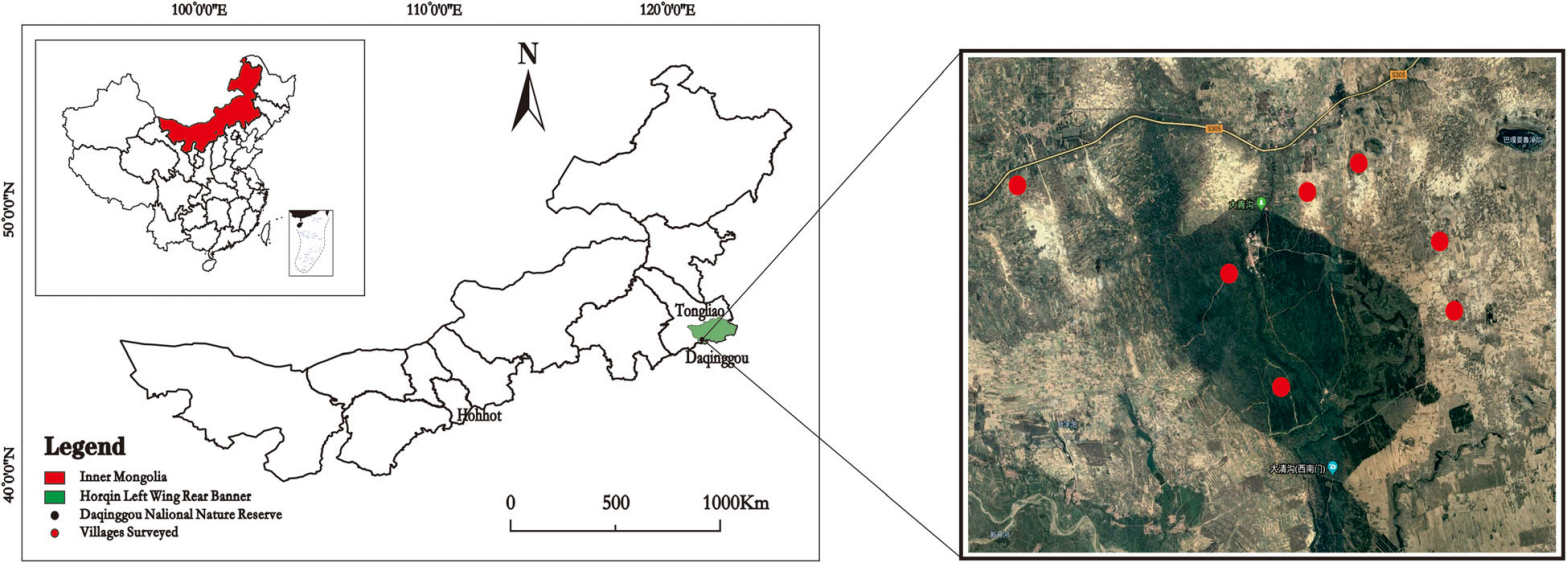
Study area and villages surveyed

Daqinggou is a national nature reserve of precious broad-leaved mixed forests, with a population of nearly 800. Locals are engaged in semifarming, semianimal husbandry, or tourism. It is a mixed area of Mongolian people and Han Chinese, with a large Mongolian population, all of whom speak Chinese. Daqinggou is located 24 km southwest of Horqin Left Wing Rear Banner, Tongliao City, Inner Mongolia Autonomous Region, longitude 120° 13′–122° 15′, north latitude 42° 45′–42° 48′ (Fig. 1), with a total area of 81.83 km^2^ and a forest area of 45.95 km^2^. The landform consists of sand dunes, sandy land and plains belonging to the subsidence zone of the Liaohe River Basin [25]. According to the “climatic regionalization of China,” it is in the transition zone from the Northeast temperate semihumid climate zone to the Inner Mongolia temperate semiarid climate zone, with an average annual temperature of 5.6 °C and mean annual rainfall of approximately 450 mm. The length of the frost-free period in the area is approximately 145 days [26].

The flora mainly consists of the flora of Changbai Mountain and Mongolian, combining the species of North China flora [27]. A total of 104 families, 320 genera, and 528 species of vascular plants grow in Daqinggou. Among them, 13 species belong to 10 different families of ferns, 3 species belong to 3 different families of gymnosperms, and 511 species belong to 91 different families of angiosperms [28].

## Methods

### Field work

**Fig. 2:**
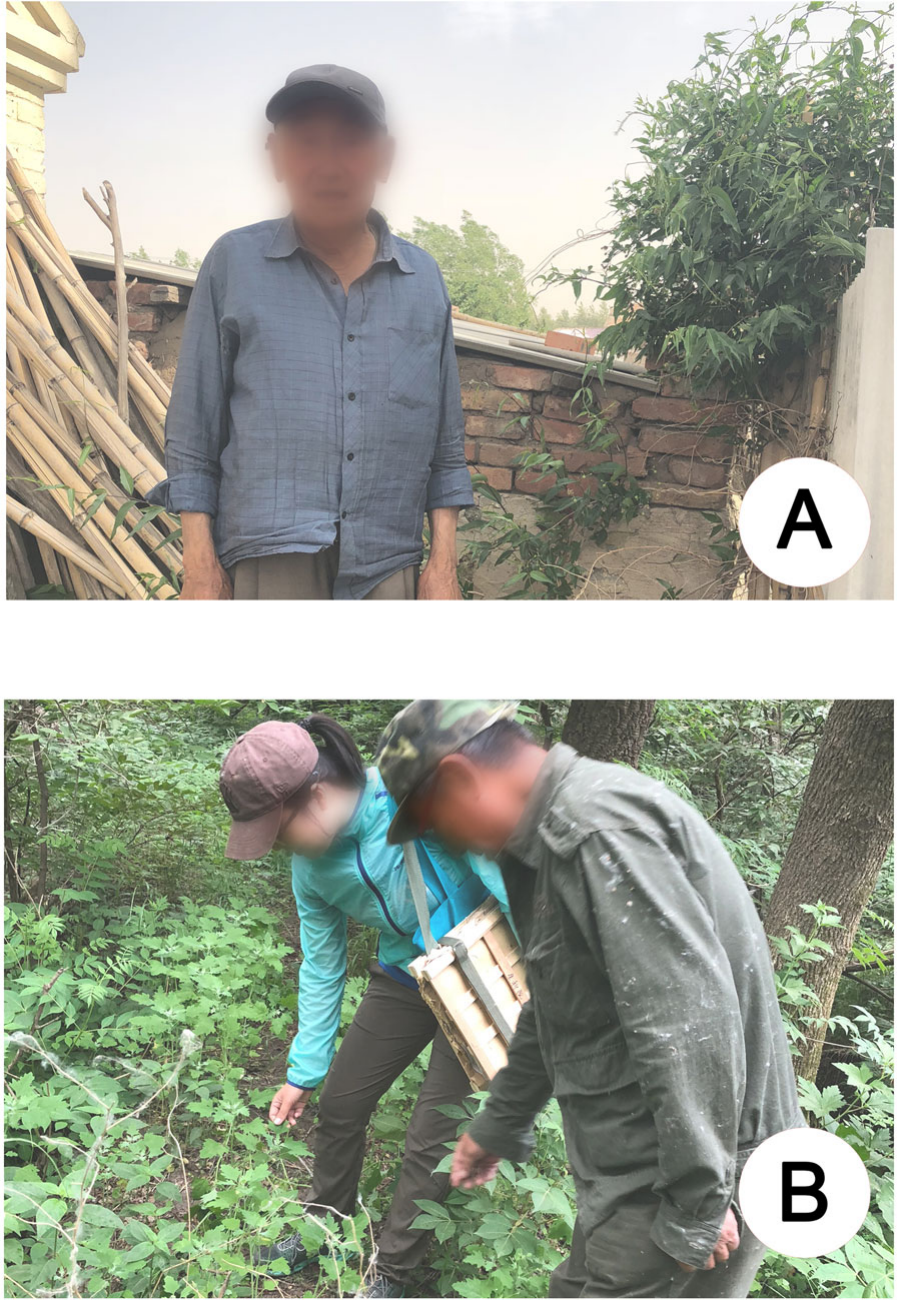
Key informant (**a**) and voucher specimen collection (**b**). Photo **a** taken by Sachula, and photo **b** taken by Manduhu

From 2017 to 2019, the authors completed 7 field studies in Daqinggou; there are two villages within the reserve and five villages in the surrounding area. Field studies included key informant interviews (Fig. 2), semistructured interviews, and questionnaires. Local farmers, retailers, and reserve staff were selected as the informants, and 227 people were interviewed. A total of 101 key informants participated in the interviews via selection using snowball sampling and intentional sampling [7, 29–32]. The ages of informants ranged from 24 to 88 (mean age 58 years), and the gender and ethnic ratios of informants were both almost 1:1 (male to female was 53 to 48, Mongolian to Han was 51 to 50). Among them, 65 people were interviewed through semistructured interviews, including questions that were relevant to document detailed information on all edible wild plants. The questions investigated included the following: What wild plants do you usually eat? Do you know any other folk name for the plant? What are the edible parts and mode of consumption of each plant?What ailment does this plant treat? Where is the habitat of the plants? Is the plant easy or difficult to collect? In 7 villages, 200 questionnaires (Additional file) about the CFSI of edible plants were distributed, and 116 valid questionnaires were collected.

### Voucher specimen

Ethnobotanical interviews and the collection of voucher specimens were carried out in two ways: locals were invited to find and collect voucher specimens of related plants at the field sites; voucher specimens were precollected and used in key informant interviews [23, 33]. A total of 84 plant specimens were aggregated as wild edible plants used by the locals. Among the collected specimens, 57 species, 3 varieties, and 1 form were identified [34, 35].

### Quantitative analysis

The CFSI was calculated to evaluate the cultural significance of wild edible plants by the following formula given by Andrea Pieroni [36]:

CFSI = QI×AI×FUI × PUI × MFF × TSAI × FMRI × 10^−2^

The formula takes into account seven indices [10, 37–40], which express the frequency of quotation (QI), availability (AI), frequency of use (FUI), plant parts used (PUI), multifunctional food use (MFFI), taste score appreciation (TSAI), and the food-medicinal role (FMRI). Based on the local cultural characteristics, the index was graded and evaluated (Table 1).

**Table 1:** Calculation and evaluation of the CFSI

Index of categories	Availability	Index value
Availability index (AI)	Very common	4.0
	Common	3.0
	Intermediate	2.0
	Rare	1.0
	Localization of the use index value	
	Ubiquitous	=
	Localized	− 0.5
	Very localized	− 0.1
Frequency of use index (FUI)	Utilization frequency	Index value
	Ordinary year	5.0
	In season	3.0
	Not used during the past 30 years	1.0
Part used index (PUI)	Part used	Index value
	Aerial parts	3
	Stems and leaves	2
	Roots, bulbs, leaves, fruits	1.5
	Bark, stems, seeds, kernel	1.0
	Flowers, inflorescence, female cone, shoots	0.75
Multifunctional food use index (MFFI)	Usage	Index value
	Raw, as snacks, cold dishes, dipped in sauce, salted	1.5
	Boiled, steamed, fried	1
	Ingredient for restricted purposes	0.75
	Condiment, grain, oil and fats	0.5
	(Usage in mixtures)	(− 0.5)
Taste score appreciation (TSAI)	Taste appreciation	Index value
	Best	10
	Good	7.5
	Fair	6.5
	Poor	5.5
	Terrible	4.0
Food-medicinal role index (FMRI)	Role as food-medicine	Index value
	Important (“that food is a medicine”, with clear specification of the treated affections)	5.0
	Intermediate (“that food is very healthy”)	3.0
	Not recognized	1.0

## Results and discussion

### Ethnobotanical inventory

There are 61 species of wild plants consumed by local people. Among them, 1 species belongs to pteridophyta, 1 species belongs to gymnosperms, and 59 species belong to 27 different families of angiosperms. This paper is summarized in the form of an ethnobotanical inventory to facilitate the analysis and evaluation. The contents of the inventory include the scientific names of species, folk Chinese and Mongolian names, usage, edible part(s) and mode of consumption, linguistic groups, and voucher numbers (Table 2).

**Table 2:** Ethnobotanical inventory of wild edible plants used by Daqinggou locals in Inner Mongolia

Species	Folk Chinese name	Folk Mongolian name	Usage	Edible part(s) and mode of consumption	Linguistic groups	Voucher numbers
*Abutilon theophrasti* Medic.	Qīng má 青麻	Him-a	Grain	Seed, dried and used as grain	M/H	D1909-001
*Adenophora remotiflora* (Sieb. et Zucc.) Miq.	Waī bó caì 歪脖菜, tǔ dǎng shēn 土党参	Uhilahu nogug-a	Vegetable/beverage	Tender leaf, fried, consumed as soup. Root, soaked with wine	M/H	D1806-032
*Adenophora polyantha* Nakai	Shā shēn 沙参	Uhilahu nogug-a	Vegetable/beverage	Tender leaf, to make soup or fried. Root, soaked with wine	M/H	D1806-036
*Allium macrostemon* Bunge	Xiǎo gēn suàn 小根蒜	Jiecong (BN), togdausu	Vegetable/condiment	Tender aerial parts and bulb, fried, dipped in sauce. Tender aerial parts, used for seasoning	M/H	D1806-011
*Allium ramosum* L.	Shān jiǔ cài 山韭菜	Heger-e in gogud	Vegetable/condiment	Tender aerial parts, fried, boiled as stuffing for pasta. Tender aerial parts and inflorescence, salted (consumed as leek flower sauce)	M/H	D1709-002
*Allium senescens* L.	Máng gé ěr (BN) 芒格尔	Manggir	Vegetable/condiment	Tender aerial parts, fried, boiled as stuffing for pasta, dipped in sauce or used for seasoning	M/H	D1806-013
*Amaranthus retroflexus* L.	Xiàn cài 苋菜	arbai	Vegetable/grain	Tender stem and leaf, consumed as soup, boiled as stuffing for pasta. Seed, dried and used as grain	M/H	D1806-003
*Armeniaca sibirica* (L.) Lam.	Shān xìng 山杏	Heger-e in guilesu	Vegetable/fruit/oil and fats	Seed, salted. Fruit, eaten raw. Kernel, oil extracted	M/H	D1805-002
*Artemisia frigida* Willd.	Xiǎo bái hāo 小白蒿	Cagan siralzi	Vegetable	Tender aerial parts, steamed with flour, consumed as soup	M/H	D1806-015
*Athyrium brevifrons* Nakai ex Kitag.	Shān jué cài 山蕨菜, lǎo yīng bǎng zi老鹰膀子	Juecai (BN), togus in segul	Vegetable	Leaf, cold and dressed with sauce, salted, fried, consumed as soup or boiled as stuffing for pasta	M/H	D1805-009
*Caltha palustris* L. var. *sibirica* Regel	Lǘ tí cài 驴蹄菜		Vegetable	Leaf, cold and dressed with sauce or fried	H	D1806-029
*Cannabis sativa* L.f. *ruderalis* (Janisch.) Chu		Olusus	Oil and fats	Seed, oil extracted	M	D1810-003
*Cerasus humilis* (Bunge) Sok.	ōu lǐ 欧李	Ulagan-a	Fruit/beverage	Fruit, eaten raw. Root, soaked with wine	M/H	D1906-001
*Chenopodium acuminatum* Willd.	Huī cài 灰菜	Gurbalzin noil	Vegetable	Tender stem and leaf, boiled as stuffing for pasta, boiled and mixed with cream	M/H	D1805-005
*Chenopodium album* L.	huī cài 灰菜	Noil	Vegetable	Tender stem and leaf, boiled as stuffing for pasta, boiled and mixed with cream	M/H	D1806-001
*Cirsium setosum* (Willd.) MB.		Cinu-a in haltar	Vegetable	Tender leaf, fried	M	D1806-014
*Codonopsis lanceolata* (Sieb. et Zucc.) Trautv.		Sun orhudai	Vegetable	Root, salted	M	D1806-025
*Corylus heterophylla* Fisch. ex Trautv.	Zhēn zi 榛子	Sid	Snack	Fruit, eaten raw, fried	M/H	D1810-002
*Crataegus pinnatifida* Bunge. var. *major* N.E. Br.	Shān lǐ hóng 山里红	Dolugun-a	Vegetable/fruit	Tender leaf, steamed with flour, consumed as soup. Fruit, eaten raw	M/H	D1805-003
*Cynanchum chinense* R. Br.		Temegen hөhu	Snack	Young fruit, eaten raw	M	D1806-006
*Cynanchum thesioides* (Freyn) K. Schum.	Lǎo piáo老瓢, lǎo guā piáo老瓜瓢	Temegen hөhu	Vegetable/snack	Young fruit, dipped in sauce, eaten raw	M/H	D1806-018
*Dendranthema indicum* (L.) Des Moul.	Yě jú huā 野菊花		Beverage	Flower, dried and made into tea	H	D1810-004
*Ephedra sinica* Stapf	Má huáng 麻黄	Zegergen-e	Fruit	Mature female cone, eaten raw	M/H	D1810-005
*Erodium stephanianum* Willd.	Hóng gēn 红根	manziuhai	Snack	Root, eaten raw	M/H	D1808-002
*Fagopyrum esculentum* Moench	Qiáo mài huā 荞麦花	Sagad	Grain	Flower, boiled and mixed with flour	M/H	D1808-001
*Ferula bungeana* Kitag.	Shān huí xiāng 山茴香		Vegetable/condiment	Tender aerial parts, boiled as stuffing for pasta, made into gruel or used for seasoning	H	D1806-019
*Hemerocallis minor* Mill.	Huáng huā zi 黄花子	Huanghuacai (BN), honghu huwar, sir-a huwar	Vegetable	Flower, consumed as soup, fried, boiled and mixed with cream	M/H	D1806-016
*Hemiptelea davidii* (Hance) Planch.		Har-a sanduu	Vegetable	Tender leaf and young fruit, consumed as soup	M	D1806-024
*Juglans mandshurica* Maxim.	Shān hé tao 山核桃	Husig-a, noyan modu	Snack	Fruit, eaten raw	M/H	D1808-003
*Kochia scoparia* (L.) Schrad.	Sào zhou cài 扫帚菜	Šugur nogo	Vegetable	Tender aerial parts, fried, steamed with flour	M/H	D1806-028
*Lespedeza davurica* (Laxm.) Schindl.		Hurbhei	Vegetable/beverage	Tender stem and leaf, fried, steamed with flour. Shoot, dried in the shade or baked and made into tea	M	D1806-030
*Lilium pumilum* DC.	Hóng huā zi 红花子, bǎi hé 百合	Saralang huwar	Vegetable	Flower, fried. Bulb, consumed as soup	M/H	D1709-001
*Lycium chinense* Mill.	Gǒu qǐ 枸杞	Gouqi (BN)	Beverage	Fruit, soaked with wine	M/H	D1806-034
*Malus baccata* (L.) Borkh.	Shān dìng zi 山定子	uril	Fruit	Fruit, eaten raw	M/H	D1805-004
*Malva verticillata* L.		Har-a nogug-a, tugur nogug-a	Vegetable	Leaf, consumed as soup, steamed with flour	M	D1806-005
*Oenanthe javanica* (Bl.) DC.	Shān qín cài 山芹菜	Cogur nogug-a	Vegetable	Stem and leaf, cold and dressed with sauce, fried, boiled as stuffing for pasta	M/H	D1806-031
*Orostachys malacophylla* (Pall.) Fisch.	Suān tǎ 酸塔	Muur in himusu	Snack	Tender aerial parts, eaten raw	M/H	D1806-023
*Padus avium* Mill.	Chòu lǐ zi 臭李子	Moil	Fruit	Fruit, eaten raw	M/H	D1805-007
*Paeonia lactiflora* Pall.	Bái shóu 白芍	Can-a	Beverage	Flower, dried and made into tea	M/H	D1906-002
*Periploca sepium* Bunge	Yáng nǎi zi 羊奶子	Imag-an eber, Šugusu modu	Vegetable/snack	Tender leaf steamed with flour. Tender fruit, eaten raw	M/H	D1806-007
*Plantago depressa* Willd.	Chē gū lu cài 车轱辘菜, chē lún cài 车轮菜	Chegulucai(BN), elzigen cihi	Vegetable	Tender leaf, steamed with flour, consumed as soup	M/H	D1806-008
*Plantago asiatica* L.	Chē gū lu cài 车轱辘菜, chē lún cài 车轮菜	Chegulucai (BN), Elzigen cihi	Vegetable	Tender leaf, steamed with flour, consumed as soup	M/H	D1806-035
*Polygonatum odoratum* (Mill.) Druce	Yù zhú 玉竹		Vegetable	Tender leaf, cold and dressed with sauce	H	D1810-007
*Polygonum aviculare* L.		Bianduya ebesu (BN)	Vegetable	Tender aerial parts, made into gruel or fried	M	D1806-027
*Polygonum divaricatum* L.	Suān bu liū 酸不溜	Simeldeg	Snack	Tender stem, eaten raw	M/H	D1806-022
*Portulaca oleracea* L.	Mǎ lián cài马莲菜, mà zha cài 蚂蚱菜	Majincai (BN)	Vegetable	Tender stem and leaf, dipped in sauce	M/H	D1806-004
*Potentilla longifolia* Willd. ex Schlecht.		Taulai in tangnai	Vegetable	Tender stem and leaf, consumed as soup or steamed with flour	M	D1806-017
*Pyrus ussuriensis* Maxim.	Shān lí 山梨	Heger-e in ilam-a	Fruit	Fruit, eaten raw	M/H	D1810-006
*Quercus mongolica* Fisch. ex Ledeb.	Xiàng zi橡子, zuó shù 柞树	Carasu	Grain/beverage/oil and fats/snack	Seed, dried and ground into flour, made wine, oil extracted or fried	M/H	D1810-001
*Salsola collina* Pall.	Zhū máo cài 猪毛菜, zhā bù leng 扎不楞	Hamhuul	Vegetable	Tender aerial parts, consumed as soup	M/H	D1806-002
*Solanum nigrum* L.		Nohai in Yзэm	Fruit/beverage	Fruit, eaten raw, soaked with wine	M	D1806-020
*Sonchus wightianus* DC.	Qǔ má cài 取麻菜	gasigun nogug-a	Vegetable/beverage	Tender stem and leaf, dipped in sauce, cold and dressed with sauce, made into tea	M/H	D1806-009
*Taraxacum mongolicum* Hand. -Mazz.	Pó pó dīng 婆婆丁	Bobodeng (BN)	Vegetable/beverage	Tender stem and leaf, dipped in sauce, cold and dressed with sauce or fried. Flower and root, dried and made into tea	M/H	D1806-010
*Thymus quinquecostatus* Cêlak. var. *asiaticus* (Kitagawa) C.Y. Wu & Y.C. Huang	Shān huā jiāo山花椒	Huajiao (BN), huaju ebesu (BN)	Condiment	Aerial parts, used for seasoning	M/H	D1806-012
*Tilia mongolica* Maxim.	Duàn shù 椴树	Domu modu	Vegetable	Tender leaf, steamed with flour, consumed as soup	M/H	D1805-006
*Ulmus macrocarpa* Hance	Yú shù 榆树	Deltu	Vegetable	Tender leaf and fruit, consumed as soup	M/H	D1805-001
*Ulmus pumila* L.	Yú shù 榆树	Hailasu	Vegetable/grain	Tender leaf and fruit consumed as soup. Bark, dried and ground into flour	M/H	D1805-008
*Urtica angustifolia* Fisch. ex Hornem.		Usun halagai	Vegetable	Tender stem and leaf, consumed as soup	M	D1806-026
*Urtica cannabina* L.	Hā lā hǎi cài (BN) 哈拉嗨菜	Halagai	Vegetable	Tender stem and leaf, consumed as soup	M/H	D1806-033
*Vitis amurensis* Rupr.	Shān pú tao 山葡萄	Heger-e in Yзэm	Vegetable/fruit/beverage	Tender leaf steamed with flour. Fruit, eaten raw, made wine	M/H	D1806-021
*Xanthoceras sorbifolium* Bunge		Sengdeng modu	Snack	Seed, eaten raw, fried	M	D1808-004

*Species in the inventory are arranged alphabetically by the plant scientific name.

*BN* borrowed name

Folk Han names of wild edible plants are written using Chinese pinyin and Chinese character names [41].

Folk Mongolian names are spelled with the Mongolian phonetic symbol [42].

### Life form characteristics of wild edible plants

The life forms of wild edible plants used by the locals are small trees, trees, shrubs, subshrubs, lianas, annual herbs, and perennial herbs. Among them, 26 species are perennial herbs and account for 43% of the total species; 12 species are annual herbs, and 11 species are trees, accounting for 20% and 18%, respectively, of the total species.

Daqinggou is a part of the steppe of Eurasia. Because of the plant distribution in the reserve, it is closely related to the broad-leaved forest of East Asia [27]. Therefore, woody plants also account for a certain proportion in this area.

### Folk names

The Mongolian people and Han Chinese living in Daqinggou are able to name most of the wild plants in their own language. Among 57 species of wild plants collected and consumed by Mongolian people, 47 species have Mongolian names, and the other 10 species directly borrow Chinese names. Among 49 species of wild plants collected and consumed by Han Chinese, *Allium senescens* is called *máng gé ěr*, and *Urtica cannabina* is called *hā lā hǎi cài*, from the plant's folk Mongolian names *manggir* and *halagai*. Therefore, the Mongolian people and Han Chinese also present exchange and reference phenomena in the nomenclature of wild edible plants.

### Food categories

The usage of wild edible plants by locals presents high diversity. According to their eating habits, food categories include grains, oils and fats, vegetables, fruits, beverages, condiments and snacks. There are 84 kinds of related plants corresponding to different usages, which is more than the total number of edible plant species, because some of the same plants have different usages (Table 3).

**Table 3:** Food category diversity

Usage	Number	Percentage
Vegetable	40	47.62
Beverage	12	14.29
Snack	10	11.90
Fruit	9	10.71
Condiment	5	5.95
Grain	5	5.95
Oil and fats	3	3.57

Among them, the wild plants used as vegetables are the most abundant. Grains were gathered and consumed during times of food shortage and famine. At present, only the bark of *Ulmus pumila* is dried and ground into flour, which is appropriately added to corn flour to increase its strength. In fact, this is a traditional way of using vegetable gum.

There are three consumption modes of wild beverage plants: Locals collect different parts of the wild plants for processing and making tea; some elders also soak the roots or fruits of wild plants in wine to make medicinal wine for drinking; *Quercus mongolica* and *Vitis amurensis* are used to make wine. Most of the wild fruits mentioned above are eaten by shepherds as snacks to satisfy hunger and thirst.

### Edible part(s) and mode of consumption

The edible parts of wild plants consumed by locals also present high diversity. According to statistics, apart from the aerial parts, the edible parts of most species are plant organs and organ combinations such as the root, stem, leaf, flower, fruit and seed, or specific parts such as the bulb, female cone, shoot, and bark. Twelve types are used. The most widely used edible parts are the fruit (17), leaf (16), and aerial parts (12) (Table 4).

**Table 4:** Diversity of edible parts

Edible part	Number	Percentage
Fruit	17	20.73
Leaf	16	19.51
Aerial parts	12	14.63
Stem and leaf	11	13.41
Flower (inflorescence)	7	8.54
Seed (kernel)	7	8.54
Root	6	7.32
Bulb	2	2.44
Bark	1	1.22
Stem	1	1.22
Shoot	1	1.22
Female cone	1	1.22

Wild edible plants are consumed in two ways, as raw food and as cooked food. The mature fruits and young fruits eaten by locals as fresh fruits and the tender stems and leaves consumed as snacks are raw food. The wild vegetables used by locals for steaming, frying, filling, soup, and seasoning plants are consumed as cooked food.

**Fig. 3:**
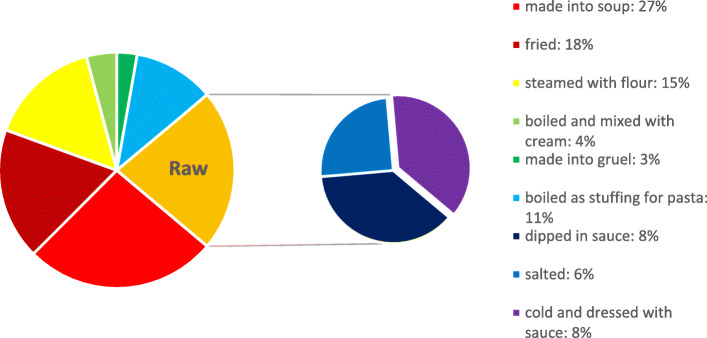
Mode of consumption of the wild vegetables

As the most widely consumed wild plants in the region, there are seven types of traditional wild vegetables (Fig. 3). Wild vegetables are frequently soaked in cold water or blanched in boiling water and then used for stir-frying or soups. For the local Mongolian people, cream is also a commonly used condiment with cooked wild vegetables. In particular, local people like to eat wild vegetables with flour and to add salt, a little oil and wild vegetables into the dough to steam a unique pasta called “bulasu”.

### Cultural food significance index (CFSI)

**Fig. 4:**
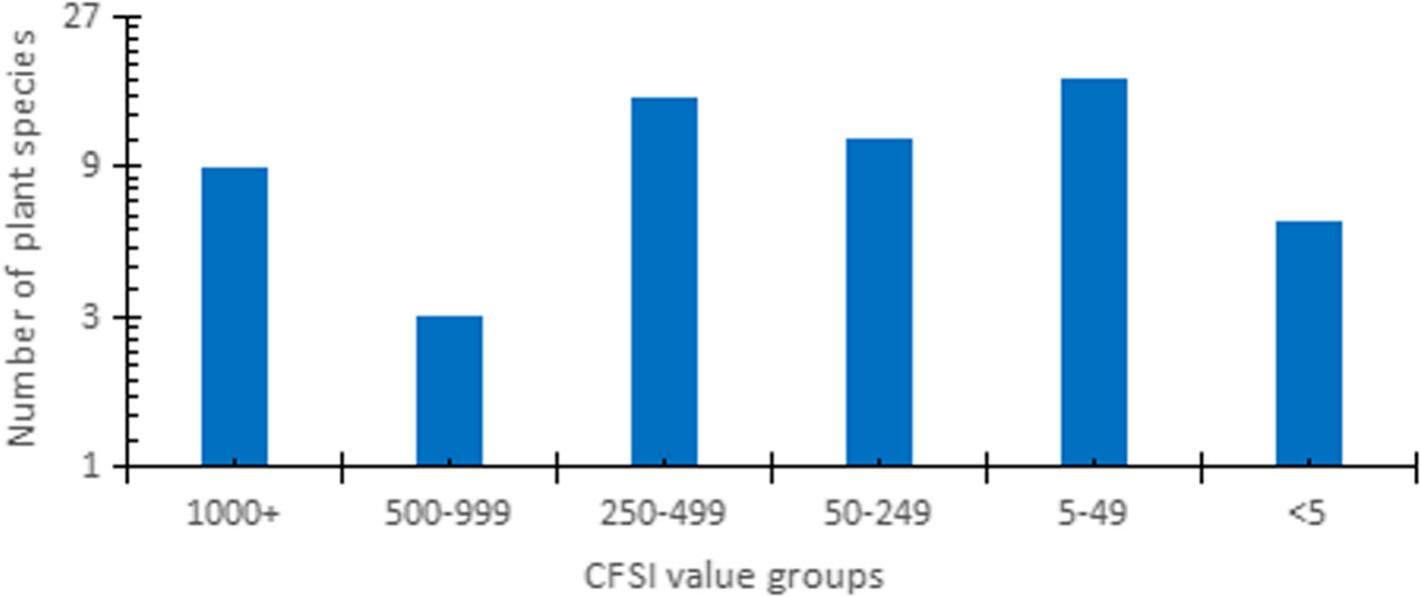
Number of plant species in each CFSI group

The CFSI values were calculated following the abovementioned formula, with a minimum of 0.04 and a maximum of 10,295.63 (Table 5). It was possible to classify the cited botanical species into six groups [36, 37]: species with very high significance (CFSI = 1000 and higher), high significance (CFSI = 500–999), moderate significance (CFSI = 250–499), low significance (CFSI = 50–249), very low significance (CFSI = 5–49), and negligible significance (CFSI < 5) (Fig. 4).

**Table 5:** Evaluation of wild edible plants of Daqinggou based on the CFSI

Species	QI	AI	FUI	PUI	MFFI	TSAI	FMRI	CFSI	Ranking
*Taraxacum mongolicum*	68.00	4.00	5.00	4.25	4.75	7.50	5.00	10295.63	1
*Crataegus pinnatifida* var. *major*	59.00	4.00	5.00	3.00	3.50	10.00	5.00	6195.00	2
*Sonchus wightianus*	75.00	4.00	5.00	2.00	3.75	7.50	5.00	4218.75	3
*Ulmus pumila*	58.00	4.00	5.00	4.00	1.50	7.50	5.00	2610.00	4
*Allium macrostemon*	44.00	2.50	3.00	4.50	3.00	10.00	5.00	2227.50	5
*Cerasus humilis*	51.00	2.50	5.00	3.00	2.25	10.00	5.00	2151.56	6
*Allium ramosum*	65.00	2.50	5.00	3.75	2.00	10.00	3.00	1828.13	7
*Armeniaca sibirica*	55.00	3.00	3.00	2.00	3.50	10.00	5.00	1732.50	8
*Oenanthe javanica*	72.00	2.50	5.00	2.00	2.00	10.00	3.00	1080.00	9
*Solanum nigrum*	36.00	3.00	5.00	1.50	2.25	7.50	5.00	683.44	10
*Plantago depressa*	46.00	4.00	3.00	1.50	2.00	7.50	5.00	621.00	11
*Allium senescens*	53.00	2.50	3.00	3.00	1.50	10.00	3.00	536.63	12
*Amaranthus retroflexus*	53.00	4.00	3.00	3.00	1.00	7.50	3.00	429.30	13
*Urtica cannabina*	47.00	4.00	3.00	2.00	1.00	7.50	5.00	423.00	14
*Vitis amurensis*	49.00	1.50	3.00	3.00	3.25	6.50	3.00	419.23	15
*Malva verticillata*	31.00	4.00	5.00	1.50	2.00	7.50	3.00	418.50	16
*Plantago asiatica*	30.00	4.00	3.00	1.50	2.00	7.50	5.00	405.00	17
*Portulaca oleracea*	49.00	4.00	3.00	2.00	1.00	6.50	5.00	382.20	18
*Ulmus macrocarpa*	46.00	4.00	3.00	3.00	1.00	7.50	3.00	372.60	19
*Quercus mongolica*	40.00	4.00	3.00	1.00	2.75	5.50	5.00	363.00	20
*Adenophora polyantha*	18.00	2.50	3.00	3.00	2.75	6.50	5.00	361.97	21
*Artemisia frigida*	51.00	2.00	3.00	3.00	2.00	6.50	3.00	358.02	22
*Cynanchum thesioides*	45.00	2.50	3.00	1.50	3.00	7.50	3.00	341.72	23
*Hemerocallis minor*	45.00	1.50	5.00	1.50	3.00	7.50	3.00	341.72	24
*Periploca sepium*	65.00	3.00	3.00	3.00	2.50	7.50	1.00	329.06	25
*Adenophora remotiflora*	14.00	2.50	3.00	3.00	2.75	6.50	5.00	281.53	26
*Potentilla longifolia*	34.00	2.50	3.00	2.00	2.00	5.50	5.00	280.50	27
*Juglans mandshurica*	47.00	2.90	3.00	1.50	1.50	10.00	3.00	276.01	28
*Salsola collina*	45.00	3.00	3.00	3.00	1.00	4.00	5.00	243.00	29
*Polygonum divaricatum*	48.00	3.00	3.00	1.00	1.50	7.50	3.00	145.80	30
*Chenopodium album*	49.00	4.00	3.00	2.00	0.50	7.50	3.00	132.30	31
*Athyrium brevifrons*	38.00	2.50	3.00	1.50	3.50	7.50	1.00	112.22	32
*Urtica angustifolia*	14.00	3.50	3.00	2.00	1.00	7.50	5.00	110.25	33
*Lespedeza bicolor*	6.00	2.50	3.00	3.75	2.75	6.50	3.00	90.49	34
*Malus baccata*	51.00	3.00	3.00	1.50	1.50	6.50	1.00	67.13	35
*Chenopodium acuminatum*	22.00	4.00	3.00	2.00	0.50	7.50	3.00	59.40	36
*Polygonatum odoratum*	9.00	2.50	3.00	1.50	1.50	7.50	5.00	56.95	37
*Dendranthema indicum*	12.00	4.00	5.00	0.75	0.75	7.50	5.00	50.63	38
*Padus avium*	34.00	3.00	3.00	1.50	1.50	6.50	1.00	44.75	39
*Lycium chinense*	13.00	1.50	5.00	1.50	0.75	6.50	5.00	35.65	40
*Ephedra sinica*	36.00	1.50	3.00	0.75	1.50	6.50	3.00	35.54	41
*Kochia scoparia*	13.00	2.00	3.00	3.00	2.00	6.50	1.00	30.42	42
*Polygonum aviculare*	2.00	2.50	3.00	3.00	2.00	6.50	5.00	29.25	43
*Pyrus ussuriensis*	52.00	1.50	3.00	1.50	1.50	5.50	1.00	28.96	44
*Lilium pumilum*	34.00	0.50	3.00	0.75	2.00	7.50	5.00	28.69	45
*Ferula bungeana*	14.00	3.00	3.00	3.00	1.00	7.50	1.00	28.35	46
*Tilia mongolica*	13.00	3.00	3.00	1.50	2.00	6.50	1.00	22.82	47
*Corylus heterophylla*	12.00	1.90	3.00	1.50	2.50	7.50	1.00	19.24	48
*Xanthoceras sorbifolium*	25.00	1.50	1.00	1.00	2.50	6.50	3.00	18.28	49
*Orostachys malacophylla*	12.00	0.50	3.00	3.00	1.50	7.50	3.00	18.23	50
*Erodium stephanianum*	35.00	2.00	3.00	1.50	1.50	0.50	5.00	11.81	51
*Cynanchum chinense*	13.00	2.00	3.00	1.50	1.50	6.50	1.00	11.41	52
*Hemiptelea davidii*	9.00	1.50	3.00	3.00	1.00	6.50	1.00	7.90	53
*Thymus quinquecostatus* var. *asiaticus*	16.00	1.50	3.00	3.00	0.50	6.50	1.00	7.02	54
*Caltha palustris* var. *sibirica*	5.00	1.90	3.00	1.50	2.50	6.50	1.00	6.95	55
*Paeonia lactiflora*	7.00	0.50	3.00	1.50	0.75	7.50	5.00	4.43	56
*Abutilon theophrasti*	14.00	3.00	1.00	1.00	0.50	4.00	5.00	4.20	57
*Cannabis sativa* f. *ruderalis*	12.00	2.50	1.00	1.00	0.50	5.50	5.00	4.13	58
*Codonopsis lanceolata*	2.00	1.50	3.00	1.50	1.50	6.50	1.00	1.32	59
*Cirsium setosum*	2.00	2.50	3.00	1.50	1.00	5.50	1.00	1.24	60
*Fagopyrum esculentum*	5.00	0.50	1.00	0.75	0.50	4.00	1.00	0.04	61

*Taraxacum mongolicum* ranks first in the CFSI, attributed to its high quotation index, multifunctional food use index value and food-medicinal role index value. Its tender stem and leaf can be used as vegetables; flowers and roots can be used for herbal drinks and have the functions of heat clearing. *Fagopyrum esculentum* ranks last with the CFSI because of the very low frequency of consumption over the past 30 years. Its flowers and a small amount of flour are cooked to satisfy hunger only in the famine years. This reported use only exists in the memory of the elderly.

### Wild edible plants used for diet therapy

In addition to their edible function, many wild edible plants also have health care functions, and a wide range of diseases can be prevented and cured by eating these plants [43–47]. The CFSI of 61 wild edible plant species shows that 27 species have medical food characteristics.

**Fig. 5:**
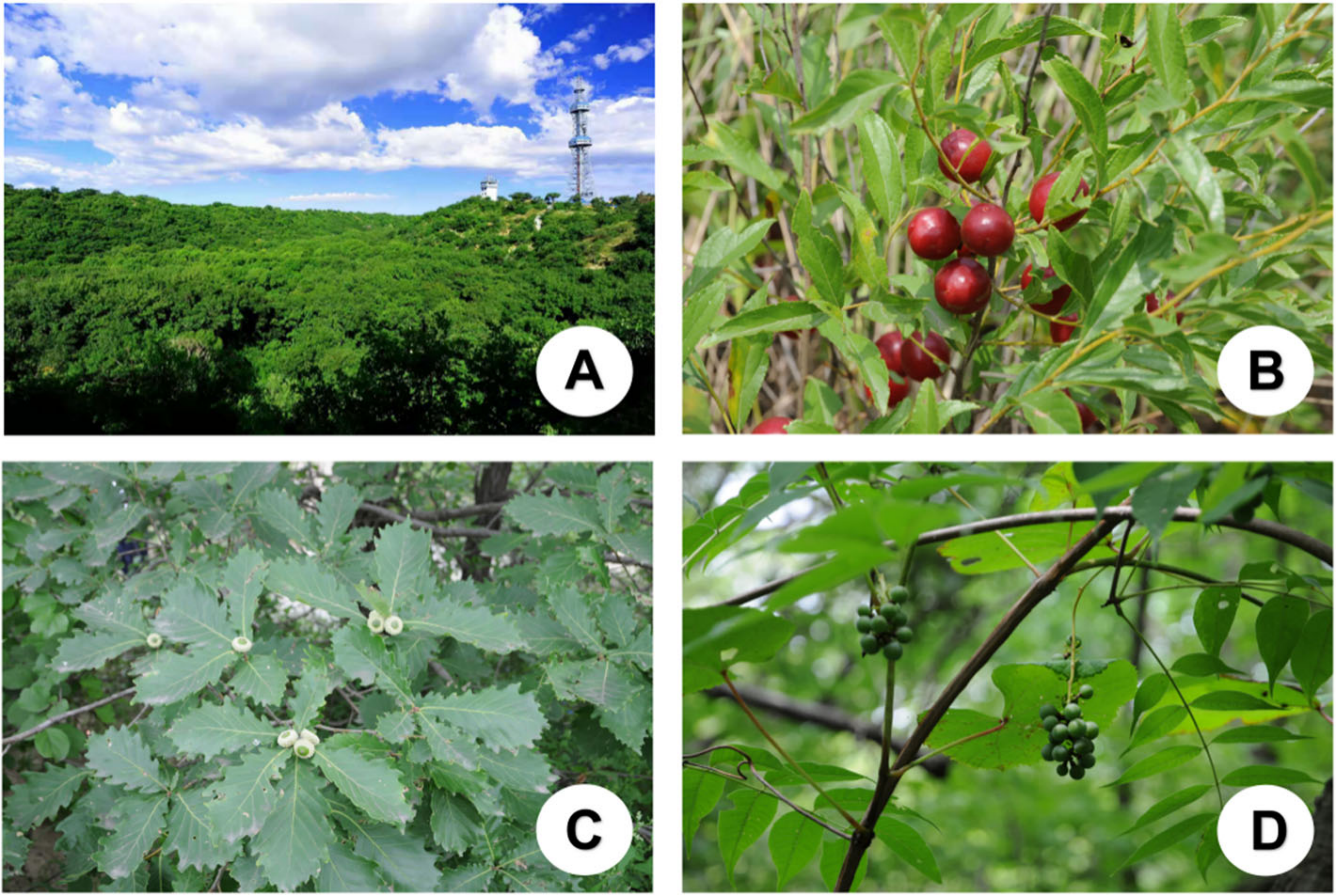
Landscape of the reserve (**a**). *Cerasus humilis* (**b**). *Quercus mongolica* (**c**). *Vitis amurensis* (**d**). **a** Photo by Manduhu, and **b**–**d** photo by Khasbagan

The locals use these plants as medicine for the following: rheumatism, diuresis, heat clearing, and tonifying Qi (Table 6). For example, patients with rheumatism can relieve their pain by drinking the medicinal wine made from the root of *Adenophora polyantha* and *Cerasus humilis*. Herbal tea made of *Sonchus arvensis* can be used for “heat clearing” according to traditional folk knowledge. A snack made from the seeds of *Quercus mongolica* can treat diarrhea in children (Fig. 5).

**Table 6:** Folk diet therapy function and the names and numbers of representative plants

Folk diet therapy function	Number	Representative plant
Treating rheumatism	6	*Abutilon theophrasti*, *Adenophora polyantha*, *Cerasus humilis*, *Erodium stephanianum*, *Urtica angustifolia*, *Urtica cannabina*
Heat clearing	4	*Dendranthema indicum*, *Polygonum aviculare*, *Sonchus wightianus*, *Taraxacum mongolicum*
Diuresis	3	*Plantago depressa*, *Plantago asiatica*, *Ulmus pumila*
Relieving cough	3	*Armeniaca sibirica*, *Lilium pumilum*, *Solanum nigrum*
Tonifying Qi	3	*Lycium chinense*, *Paeonia lactiflora*, *Polygonatum odoratum*
Heat clearing and detoxifying	2	*Portulaca oleracea*, *Potentilla longifolia*
Promoting digestion	2	*Allium macrostemon*, *Crataegus pinnatifida* var. *major*
Relaxing the bowels	2	*Adenophora remotiflora*, *Cannabis sativa* f. *ruderalis*
Lowering blood pressure	2	*Portulaca oleracea*, *Salsola collina*
Reducing blood glucose	1	*Polygonatum odoratum*
Relieving diarrhea	1	*Quercus mongolica*
Improving eyesight	1	*Lycium chinense*

## Conclusions

A total of 61 species of wild plants are consumed by local people. Among them, *Abutilon theophrasti*, *Fagopyrum esculentum*, and *Lycium chinense* have been reduced to escaped species, common along roadsides and in wasteland and fields. A total of 126 folk names of local wild edible plants were recorded. Among them, the Mongolian people provided 67 folk names, corresponding to 57 wild plants, and the Han Chinese provided 58 folk names, corresponding to 49 wild plants. The corresponding rates of Mongolian and Han folk names and scientific names were 85.07% and 84.48%, respectively, which indicates that scientific knowledge has high consistency with traditional knowledge in botanical nomenclature [48–50]. However, the choice of edible parts and consumption between the two linguistic groups was almost identical. Based on the statistics, the most widely eaten parts of wild edible plant species are aerial parts, stems, and leaves. Wild edible plant species are most commonly cooked (as a soup) or eaten fresh.

The CFSI of wild edible plant species was also calculated, with a minimum of 0.04 and a maximum of 10295.63. *Taraxacum mongolicum*, a local common natural potherb, was the most popular wild edible plant based on its high CFSI metric. According to the corresponding records of Chinese herbs and Mongolian medicine [51–55], among the 27 species of wild diet therapy plants, 19 species were recorded based on Mongolian medicine, and 23 species were recorded based on Chinese herbal medicine. *Adenophora remotiflora*, *Cannabis sativa* f. *ruderalis*, and *Salsola collina* have not been recorded in the relevant literature. Twelve species have been recorded in the literature of “Chinese Materia Medica: Mongolian Medicine roll”, and 23 species have been recorded in the literature of “Planta medica records of Inner Mongolia”. This reflects that the locals have high consistency with Mongolian medicine and Chinese herbal medicine in the selection of wild diet therapy plants; 17 species have been recorded in the literature of “Pharmacopoeia of the People’s Republic of China”, which shows that the knowledge of local wild diet therapy plants has a strong scientific basis and is worth further study.

## Supplementary information

**Additional file 1.** Questionnaires.

## Data Availability

All data generated or analyzed during this study are included in this published article and its supplementary information files.

## References

[1] Xue DY (2014). International process of protection of traditional knowledge under the Convention on Biological Diversity. Guizhou Soc Sci..

[2] Natcher D, Kalagnanam V, Rawal R, Johnston M, Mamun AA (2017). Non-timber forest products and village livelihoods in Rajasthan, India: adaptation in a changing environment. Int J Sust Dev World..

[3] Meke GS, Mumba RFE, Bwanali RJ, Williams VV (2017). The trade and marketing of traditional medicines in southern and central Malawi. Int J Sust Dev World..

[4] Dzerefos CM, Witkowski ETF, Kremerköhne S (2016). Aiming for the biodiversity target with the social welfare arrow: medicinal and other useful plants from a critically endangered grassland ecosystem in Limpopo Province, South Africa. Int J Sust Dev World..

[5] Ma Y, Luo BS, Wen Q, Feng JC, Xue DY (2019). A case study on traditional knowledge and the diversity of edible plants use from the ecological immigrants with different sources in Hongsibu district of Ningxia Autonomous Region. J Plant Gen Resour..

[6] Pei SJ (2013). Ethnobotany and the sustainable use of biodiversity. Plant Divers Resour..

[7] Pei SJ, Huai HY (2007). Ethnobotany.

[8] Cotton CM (1996). Ethnobotany: principles and applications.

[9] Song ML (2011). Wild edible compositae resources in Lishan National Nature Reserve of Shanxi. Chin Wild Plant Resour..

[10] Geng Y, Zhang Y, Wang YH (2016). Traditional knowledge and its transmission of wild edibles used by the Naxi in Baidi village. Northwest Yunnan Province. J Ethnobiol Ethnomed..

[11] Luo BS, Liu B, Zhang HZ, Zhang HK, Li X, Ma LJ, Wang YZ, Bai YJ, Zhang XB, Li JQ, Yang J, Long CL (2019). Wild edible plants collected by Hani from terraced rice paddy agroecosystem in Honghe Prefecture, Yunnan. China. J Ethnobiol Ethnomed..

[12] Yan J, Zhuo JX, Luo BS, Long CL (2013). Eating from the wild: diversity of wild edible plants used by Tibetans in Shangri-la region, Yunnan. China. J Ethnobiol Ethnomed..

[13] Hong LY, Zhuo JX, Lei QY, Zhou JJ, Ahmed S, Wang CY, Long YX, Li FF, Long CL (2015). Ethnobotany of wild plants used for starting fermented beverages in Shui communities of southwest China. J Ethnobiol Ethnomed..

[14] He JW, Zhang RF, Lei QY, Chen GX, Li KG, Ahmed S, Long CL (2019). Diversity, knowledge, and valuation of plants used as fermentation starters for traditional glutinous rice wine by Dong communities in Southeast Guizhou. China. J Ethnobiol Ethnomed..

[15] Khasbagan HHY, Pei SJ (2000). Wild plants in the diet of Arhorchin Mongol herdsmen in inner Mongolia. Econ Bot..

[16] Wujisguleng W, Khasbagen K (2010). An integrated assessment of wild vegetable resources in Inner Mongolian Autonomous Region. China. J Ethnobiol Ethnomed..

[17] Li S, Zhang Y, Guo Y, Yang L, Wang Y (2020). Monpa, memory, and change: an ethnobotanical study of plant use in Mêdog County, South-east Tibet. China. J Ethnobiol Ethnomed..

[18] Kang Y, Łuczaj Ł, Kang J, Zhang S (2013). Wild food plants and wild edible fungi in two valleys of the Qinling Mountains (Shaanxi, central China). J Ethnobiol Ethnomed..

[19] Kang Y, Łuczaj Ł, Yes S, Zhang S, Kang J (2012). Wild food plants and wild edible fungi of Heihe valley (Qinling Mountains, Shaanxi, Central China): herbophilia and indifference to fruits and mushrooms. Acta Soc Bot Pol..

[20] Kang YX, Łuczaj Ł, Kang J, Wang F, Hou JJ, Guo QP (2014). Wild food plants used by the Tibetans of Gongba Valley (Zhouqu county, Gansu, China). J Ethnobiol Ethnomed..

[21] Kang J, Kang YX, Ji XL, Guo QP, Jacques G, Pietras M, Łuczaj N, Li DW, Łuczaj Ł (2016). Wild food plants and fungi used in the mycophilous Tibetan community of Zhagana (Tewo County, Gansu, China). J Ethnobiol Ethnomed..

[22] Khasbagan Y (2011). Zhao H. Study on traditional knowledge of wild edible plants used by the Mongolians in Xilingol typical steppe area. Plant Divers Resour..

[23] Man L, Zhang XS (2007). Khasbagan, Erdemtuu. Study on the Mongolian traditional knowledge of wild edible plants in Ordos plateau. Acta Bot Yunnanica..

[24] Khasbagan, Soyolt, Man L, Enhebayar, Gerelt, Hu WR. Traditional usage of wild plants for food by the Ejina Mongolians and its exploitation and ethnoecological significance. J IMNU (Natural Science Edition). 2005; 34(4): 471-4, 88. 10.3969/j.issn.1001-8735.2005.04.020.

[25] Liu GF, Bai L, Liu YP, Cheng WY, Dafubaiyila, Zheng Q F. Species diversity of phytocenosis at the upper slopes in Daqinggou Nature Reserve. J IMUN (Nat Sci Edit).. 2016; 31(4): 293-7, 369. 10.14045/j.cnki.15-1220.2016.04.005.

[26] Zheng YR (1999). Main woody species niche of plant community in Daqinggou. Acta Phytophy Sin..

[27] Ma YQ (1998). Flora of Inner Mongolia, vol. I. 2nd ed.

[28] Writing committee of “Annals of Horqin Left Wing Rear Banner”. Annals of Horqin Left Wing Rear Banner. Hulun Buir: Inner Mongolia Culture Press; 2008.

[29] Pei SJ, Long CL. Applied ethnobotany. Kunming, Yunnan, China: the Nationalities Publishing House of Yunnan; 1998.

[30] Martin GJ (1995). Ethnobotany: a methods manual.

[31] Harshberger JW (1896). The purpose of ethno-botany. Bot Gaz..

[32] Wang YH, Wang C. Ethnobotany: common research methods: Zhejiang Education Publishing House; 2017.

[33] Wurchaih, Huar, Menggenqiqig, Khasbagan. Medicinal wild plants used by the Mongol herdsmen in Bairin Area of Inner Mongolia and its comparative study between TMM and TCM. J Ethnobiol Ethnomed. 2019; 15:32. 10.1186/s13002-019-0300-9. 10.1186/s13002-019-0300-9 PMC6609360 31269968

[34] Ma YQ (1990). Flora of Inner Mongolia, vol. 2. 2nd ed.

[35] Flora of China Editorial Committee. Flora of China. Beijing: Science Press, and St. Louis: Missouri Botanical Garden Press; 1994-2013.

[36] Pieroni A (2001). Evaluation of the cultural significance of wild food botanicals traditionally consumed in Northwestern Tuscany. Italy. J Ethnobiol..

[37] Suiarwo W, Caneva G (2016). Using quantitative indices to evaluate the cultural importance of food and nutraceutical plants: comparative data from the Island of Bali (Indonesia). J Cult Herit..

[38] Yang NT, Zhang Y, He LJ, Fan RY, Gou Y, Wang C, Wang YH (2018). Ethnobotanical study on traditional edible sour plants of Bai nationality in Dali area. J Plant Resour Envir..

[39] Turner NJ (1988). “The importance of a rose”: evaluating the cultural significance of plants in Thompson and Lillooet Interior Salish. American Anthropologist..

[40] Tardío J, Pardo-de-Santayana M (2008). Cultural importance indices. a comparative analysis based on the useful wild plants of southern Cantabria (Northern Spain). Econ Bot..

[41] Dictionary Office of Language Research Institute, Chinese Academy of Social Sciences. Modern Chinese dictionary. Commercial Press; 2016.

[42] Mongolian dictionary Committee. Mongolian dictionary. Hohhot: Inner Mongolian People’s Press; 1997.

[43] Zhang LL, Zhang Y, Pei SJ, Geng YF, Wang C, Wang YH (2015). Ethnobotanical survey of medicinal dietary plants used by the Naxi People in Lijiang Area, Northwest Yunnan. China. J Ethnobiol Ethnomed..

[44] Khasbagan. Wild plants used for the folk dietotherapy in Arhorchin Mongolians. J Chin Med Mat. 2001;24(2):83–85. 10.3321/j.issn:1001-4454.2001.02.003. 11402735

[45] Shang YB (2009). Sun LH.

[46] Pei SJ (2007). Brief discussion on ethno-medicine research and new-drug development of China (Part A). J Yunnan Univ Tradit Chin Med..

[47] Pei SJ (2007). Brief discussion on ethno-medicine research and new-drug development of China (part B). J Yunnan Univ Tradit Chin Med..

[48] Khasbagan, Soyolt. Indigenous knowledge for plant species diversity: a case study of wild plants' folk names used by the Mongolians in Ejina desert area, Inner Mongolia, P. R. China. J Ethnobiol Ethnomed. 2008; 4: 2. 10.1186/1746-4269-4-2. 10.1186/1746-4269-4-2 PMC2268917 18199323

[49] Soyolt, Galsannorbu, Yongping, Wunenbayar, Liu GH, Khasbagan. Wild plant folk nomenclature of the Mongol herdsmen in the Arhorchin national nature reserve, Inner Mongolia, PR China. J Ethnobiol Ethnomed. 2013; 9: 30. 10.1186/1746-4269-9-30. 10.1186/1746-4269-9-30 PMC3649933 23628479

[50] Yeruhan K (2008). Twenty species of wild plants were named and used by the Mongol herdsman in Bolgan Som, Bayandelger Gacha, Xilinhot. J IMNU (Nat Sci Edit)..

[51] Zhu YM (2000). Planta medica records of Inner Mongolia.

[52] Zhu YM (1989). Planta medica records of Inner Mongolia.

[53] Zhu YM (1989). Planta medica records of Inner Mongolia.

[54] China State Administration of Traditional Chinese Materia Medica Committee (2004). Chinese materia medica: Mongolian drugs.

[55] China Pharmacopoeia Commission. Pharmacopoeia of the People's Republic of China. Beijing: China Medical Science Press; 2010.

