# Supplementary material for: Wild edible plants collected and consumed by the locals in Daqinggou, Inner Mongolia, China
Source: J Ethnobiol Ethnomed. 2020 Oct 9;16:60. doi: 10.1186/s13002-020-00411-2 (PMC7547461; doi:10.1186/s13002-020-00411-2)
Supplement: Supplementary file 1 — Additional file 1. Questionnaires. [file 13002_2020_411_MOESM1_ESM.docx]

| Investigation site： village | | | |
| --- | --- | --- | --- |
| Investigation time (year/month/day)： / / | | | |
| Name： Minority： Gender： Age： Occupation： degree of education： | | | |
| **Utilization of edible plants**（Please fill in the attached table carefully according to the actual situation）   1. Which wild plants can be used as vegetables? 2. Which wild plants can be used as fruits, seasoning? 3. Which wild plants can be used as tea, grain? 4. In addition to the above purposes, there are other uses of edible plants? | | | |
| **Attached table ( One table, one plant )** | | | |
| Folk plant name: | | | |
| Edible parts |  | | |
| Food categories |  | | |
| Mode of consumption |  | | |
| Availability | Very common | Food-Medicinal Role | High (“that food is a medicine”, with clear specification of the treated affections) |
|  | Common |  |  |
|  | Middle |  | Middle high (“that food is very healthy”) |
|  | Rare |  |  |
| Habitat | Ubiquitous |  | Not recognized |
|  | Localized | Taste Score Appreciation | Best |
|  | Very localized |  | Good |
| Frequency of Use | Ordinary year |  | Fair |
|  | Be in season |  | Poor |
|  | No longer used During the past 30 years |  | Terrible |

**Questionnaires**
